# The uterine adenosarcoma with postoperative residual in a woman treated by total abdominal hysterectomy/bilateral salpingo‐oophorectomy: A case report and review of literature

**DOI:** 10.1002/cnr2.1891

**Published:** 2023-08-17

**Authors:** Pedram Fadavi, Maryam Garousi, Sepideh Soltani, Fatemeh Montazer, Maryam Abolhasani, Salar Asgari, Elahe Mirzaee

**Affiliations:** ^1^ Department of Radiation Oncology, School of Medicine Iran University of Medical Sciences Tehran Iran; ^2^ Department of Pathology, School of Medicine Iran University of Medical Sciences Tehran Iran; ^3^ Oncopathology Research Center, School of Medicine Iran University of Medical Sciences Tehran Iran

**Keywords:** adenosarcoma, case report, total abdominal hysterectomy, uterine

## Abstract

**Background:**

In patients with uterine adenosarcoma, a total abdominal hysterectomy (TAH) with bilateral salpingo‐oophorectomy (BSO) is typically recommended as an initial treatment. There is no consensus on adjuvant therapies.

**Case:**

We report the case of a patient with uterine adenosarcoma with postoperative residual disease. We performed four courses of adjuvant chemotherapy, including Ifosfamide, Mesna, and Adriamycin, and whole pelvic radiation with a dose of 50.4 Gy/28 Fr.

**Conclusion:**

A combination of chemotherapy and radiotherapy may be a promising treatment option for uterine adenosarcoma with postoperative residual disease.

## 1 INTRODUCTION

Clement and Scully initially reported uterine adenosarcoma (UA) in 1974 as Mullerian adenosarcoma, a mixed uterus tumor consisting of benign glandular epithelium and malignant mesenchymal tissue. Uterine adenosarcoma is a rare malignancy that makes up about 8% of all uterine sarcomas and 0.2% of malignant uterine tumors.^1^ Most commonly, this tumor is formed from the endometrium.^2^, ^3^ Several risk factors influence the prognosis and survival rates of patients with such diseases, including sarcomatous overgrowth, lymphovascular space invasion, and deep myometrial invasion.^4^, ^5^ Older age at diagnosis is a risk factor associated with worse progression‐free survival (PFS) and cardiovascular disease associated with worse overall survival (OS).^4^ There are no genetic mutations associated with uterine sarcomas, but childhood retinoblastoma and renal cell carcinoma syndrome have been connected to sarcomas of the uterus.^6^, ^7^, ^8^

The current standard of care for patients with uterine adenosarcoma is total abdominal hysterectomy (TAH) with bilateral salpingo‐oophorectomy (BSO).^4^ Undoubtedly, there is disagreement on other aspects of such therapy, including surgical treatment (oophorectomy or lymphadenectomy)^7^, ^9^ and adjuvant therapies (chemotherapy or radiotherapy).^4^

The case report discusses a woman with a uterine adenosarcoma detected following a total abdominal hysterectomy and bilateral salpingo‐oophorectomy with residual disease in the vaginal cuff.

## 2 CASE REPORT

A 63‐year‐old woman with two pregnancies—both normal vaginal delivery (NVD)—and menopause at age 50 years, referred to Hafte Tir Hospital (Tehran, Iran) on August 2021 due to a 6‐month history of abdominal pain and vaginal spotting.

There was no particular disease in the family or personal history. The patient was evaluated by transabdominal sonography of the uterus and ovaries, which showed a mass of 55 × 29 mm^2^ on the endometrial cavity in favor of uterus myoma. The laboratory investigation of Cell blood counts (WBC: 4.5 10^3^/μL, Hb: 11 g/dL, Plt: 250 10^3^/mm^3^ and other basic tests related to the liver (AST: 15 U/L, ALT: 19 U/L, ALP: 115 IU/L, Total Billirubin: 1.08 mg/dL and kidney (urea: 36 mg/dL, Creatinine: 0.6 mg/dL) were reported.

**FIGURE 1:**
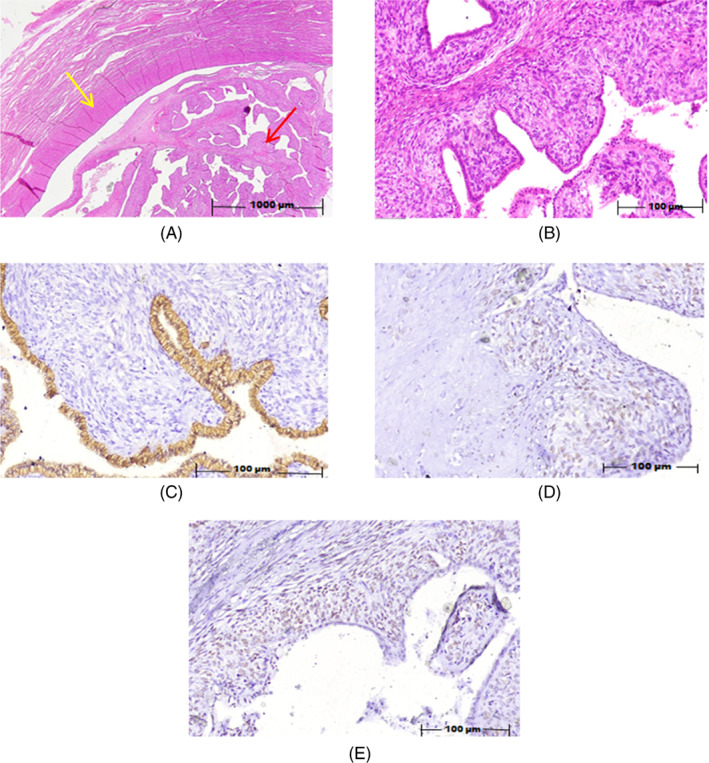
(A) H&Ex2: a biphasic tumor with intimately admixed glands and sarcomatous stroma. Intraglandular growth with projections narrowing the glandular lumens and a leaf‐like (phyllodes‐like) architecture (The red arrow) and without myometrial invasion (The yellow arrow). (B) H&Ex20: Periglandular stromal condensation (cuffing). (C) IHC studyx20 for pan‐CK: Glandular component, with positive staining. (D) IHC studyx20 for ER: Mesenchymal component with weak to moderate nuclear positive staining. (E) IHC studyx20 for P53: Mesenchymal component with weak to moderate nuclear positive staining. [Correction added on 22 September 2023, after first online publication: Figure 1 and its legend have been updated in this version]

A hysterectomy was done due to severe bleeding. A polypoid tumoral lesion measuring 7.5 cm in the postoperative gross pathology was observed, which occupied the endometrial cavity. The microscopic examination showed a fungating mass composed of hypercellular endometrial‐like stroma, covered by a single layer of flattened cells forming large numbers of polypoid projections. The stroma was mildly hypercellular with condensation around the surface epithelium. Areas of stromal edema were also seen. Mild nuclear pleomorphism of stromal spindle cells was seen. Mitotic figures were estimated at 4–5/10 hpf. No stromal invasion was identified (Figure 1A,B). Immunohistochemical staining was performed to confirm the diagnosis. The IHC study was positive for Panck, SMA, CD10, Desmin, ER, PR, and P53 and negative for WT1 (Figure 1C–E). According to these findings, the diagnosis was compatible with Mullerian adenosarcoma of the endometrium, with no myometrial invasion and positive ki76 staining in about 10% of neoplastic stromal components.

**FIGURE 2:**
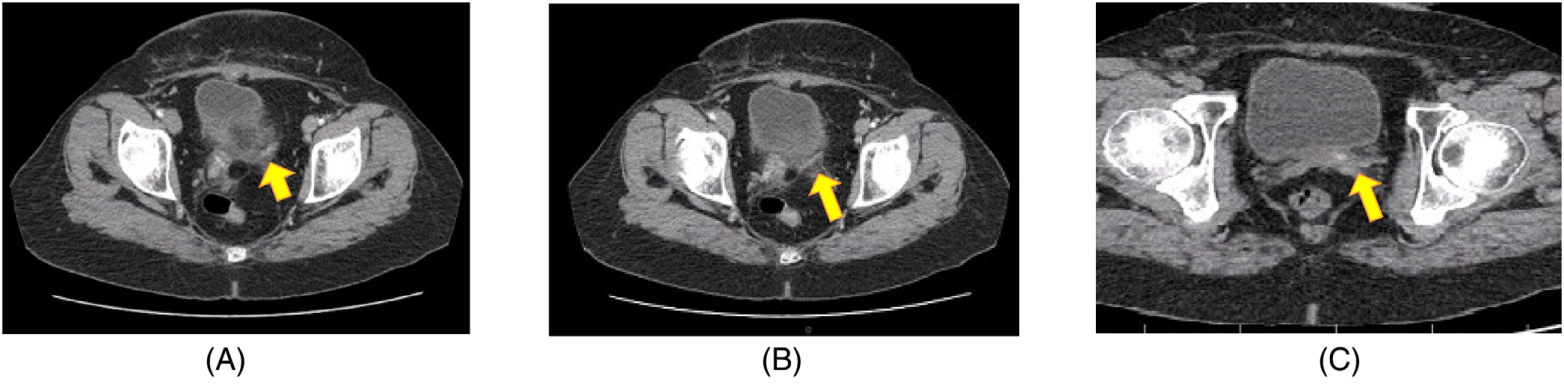
CT scan of the abdomen and the pelvis with and without injected contrast, axial plane 1 month after TAHBSO surgery was provided. The yellow arrow show a hypodense mass in the vaginal cuff (A: At the level of acetabulum. B: At the level of top of femoral head bone. C: At the level of femoral head bone).

One month after TAHBSO surgery, a computed tomography scan (CT scan) of the chest, the abdomen, and the pelvis with and without contrast injection was done. Soft tissue was observed in the vaginal cuff in the abdominal and pelvic CT (Figure 2).

**FIGURE 3:**
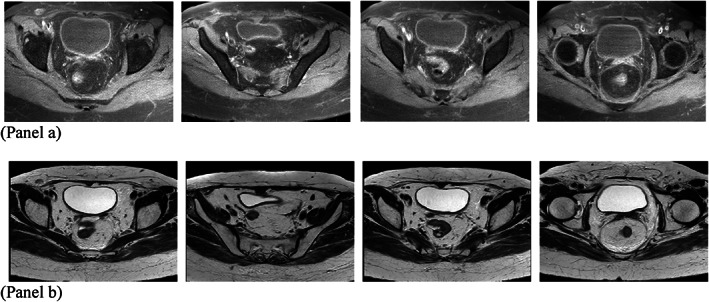
MRI of the abdomen and the pelvis with injected contrast, after 9 months of treatment with normal feature and no sign of tumor recurrence. (Panel A: T1‐weighted sequence FAT SAT. Panel B: T2‐weighted sequence).

Regarding the inoperable residue, the patient underwent four courses of adjuvant chemotherapy (Ifosfamide, Mesna, Adriamycin), and after 1 month, she underwent whole pelvic radiation with a dose of 50.4 Gy/28 fr. 3 months after the completion of treatment, the patient underwent Pelvis MRI with and without GAD, which showed a complete response. Currently, the patient has no evidence of recurrence or metastasis after 9 months of treatment (Figure 3).

## 3 DISCUSSION

This case report describes a postmenopausal patient with uterine adenosarcoma and postoperative residue. She underwent TAH with BSO and adjuvant chemotherapy and radiotherapy without recurrence, metastasis invasion, or sarcomatous overgrowth after 9 months of follow‐up.

It has been reported that uterine sarcomas represent less than 1% of gynecological cancers and 3%–7% of all uterine cancers.^10^ Uterus sarcomas are heterogeneous cancers with similar pathological and clinical features.^11^ An endometrial adenosarcoma begins as a polyp on the surface of the endometrium and then invades the myometrium. They often have low malignant potentials. Relapse rates are about 24% in adenosarcomas, and the extent of myome‐trial invasion has been reported to be an important factor in the risk of relapse.^12^, ^13^ Menstrual disorders and abnormal vaginal bleeding are the most common symptoms of this disease.^14^

MRI is helpful in the diagnosis of uterine adenosarcoma.^15^, ^16^, ^17^ Histopathologically, adenosarcoma consists of benign glands as well as low‐grade stroma. In most cases, there is always immunoreactivity for ER, PR, and CD10. Markers associated with mesenchymal cells, such as SMA, desmin, and CD34, can be positive in stromal cells. There is always a negative cytokeratin expression in the sarcomatous component.^18^ Uterine adenosarcoma is usually a low‐grade tumor with a good prognosis, unlike other high‐grade uterine sarcomas with high‐risk features for recurrences and metastases.^19^, ^20^

Among uterine adenosarcomas, myometrial invasion is regarded as an important histopathological prognostic factor.^21^, ^22^ It was found that the recurrence rate for adenosarcoma with myometrial invasion was 36%, compared with 7% for tumors without myoinvasion. The overall survival rate for tumors with myoinvasion was about 60%, while the rate for tumors with associated metastases was less than 50%.^23^ Lymph node metastases are rare in uterine adenosarcoma, which means a worse prognosis if they occur. Earlier studies have linked tumor size, myometrial invasion, and sarcomatoid overgrowth to lymph node metastases.^24^

Univariate analyses of Seagle's study showed that older patients had higher mortality risks.^19^

A soft tissue sarcoma is usually surgically resected with appropriately negative margin,^25^ while uterine adenosarcoma patients undergo bilateral salpingo‐oophorectomy and total hysterectomy.^7^ There is limited data on the efficacy of ovarian preservation in uterine adenosarcomas.^26^ It is difficult to individualize uterine adenosarcoma from benign polyps and adenofibroma.^27^ For a correct diagnosis, a hysterectomy is necessary to remove the entire tumor and examine it.^15^

The combination of TAH and BSO is accepted as standard treatment by most physicians.^4^ Other therapeutic aspects remain controversial, such as the necessity of performing oophorectomy or lymphadenectomy^9^, ^28^ or using adjuvant chemotherapy or radiotherapy.^4^ There is also no consensus on adjuvant therapies. It is also believed that such treatments are associated with a longer survival time and reduced mortality of patients with advanced stage, deep myometrial invasion, presence of vascular invasion, sarcomatous overgrowth, as well as adjuvant therapy.^4^

Early‐stage uterine adenosarcoma patients have a good prognosis if some risk factors are not present, but if any of these factors are present, such as deep myometrial invasion, presence of vascular invasion, and sarcomatous overgrowth, survival rates are lower.^4^ A sarcoma‐based regimen contains Ifosfamide and Doxorubicin as adjuvant chemotherapy.^29^ Some series have shown the role of radiotherapy in the local control.^4^, ^30^ There have been some cases of uterine adenosarcoma in the English literature^29^, ^31^, ^32^, ^33^, ^34^, ^35^; these are summarized in Table 1.

**TABLE 1:** Published studies of uterine adenosarcoma.

Case	References	Age (Years)	High risk features	Adjuvant therapy
1	Shahidsales et al.^29^	24	Sarcomatous overgrowth	Chemotherapy + Radiotherapy
2	Kandaz et al.^31^	39	Sarcomatous overgrowth	Chemotherapy
3	Qiyue et al.^32^	38	Myometrial invasion	Chemotherapy
4	Goh et al.^33^	21	Without risk factor	Without Adjuvant Therapy
5	Zaloudek et al.^34^	15	Myometrial invasion	Radiotherapy
6	Garcia‐Mendoza et al.^35^	46	Sarcomatous overgrowth	Without Adjuvant Therapy

Uterine adenosarcoma is a rare cancer, so there is no consensus on adjuvant therapies. In this case report, with the residual disease in the vaginal cuff and sarcomatous overgrowth, adjuvant chemotherapy and radiotherapy were considered despite the absence of other high‐risk factors in the pathology. Unlike many studies that chose only one modality, we chose both treatment modalities due to the presence of residual disease, which was not seen in other similar studies.

## 4 CONCLUSION

This case report describes a postmenopausal patient with uterine adenosarcoma. Following TAH with BSO and adjuvant chemotherapy and radiotherapy, the subject was reported to be free of recurrence or metastasis, with no evidence of sarcomatous overgrowth after 9 months of follow‐up. The results obtained from this patient might address the role of adjuvant therapies in treating patients with the residual disease. Obtaining more information, like this case, will help treat this rare condition.

## AUTHOR CONTRIBUTIONS

**Pedram Fadavi:** Conceptualization (equal); methodology (equal). **Maryam Garousi:** Software (equal); supervision (equal). **Sepideh Soltani:** Resources (equal); writing – original draft (equal). **Fatemeh Montazer:** Formal analysis (equal). **Maryam Abolhasani:** Data curation (equal). **Salar Asgari:** Conceptualization (equal); data curation (equal). **Elahe Mirzaee:** Conceptualization (equal); methodology (equal); supervision (equal); writing – review and editing (equal).

## CONFLICT OF INTEREST STATEMENT

The authors declare no conflicts of interest.

## ETHICS STATEMENT

We obtained a written statement of informed consent from the patient for the publication of case details and use of images. The case discussed in this manuscript does not include patient‐identifying information, nor does it report a new study that required IRB approval.

## Data Availability

The data that support the findings of this study are available from the corresponding author upon reasonable request.
